# Geriatric evaluation and management inpatients spend little time participating in physically, cognitively or socially meaningful activity: a time–motion analysis

**DOI:** 10.1093/ageing/afaf043

**Published:** 2025-03-04

**Authors:** Laura Jolliffe, Taya A Collyer, Ka Hei Sun, Lisa Done, Siobhan Barber, Michele L Callisaya, David A Snowdon

**Affiliations:** Department of Occupational Therapy, School of Primary and Allied Health Care, Monash University, Peninsula Campus, 47-49 Moorooduc Highway, Frankston, Victoria, 3199, Australia; Peninsula Health, 2 Hastings Road, Frankston, Victoria, 3199, Australia; National Centre for Healthy Ageing, Ngarnga Centre, 2 Hastings Road, Frankston, Victoria, 3199, Australia; Peninsula Health, 2 Hastings Road, Frankston, Victoria, 3199, Australia; National Centre for Healthy Ageing, Ngarnga Centre, 2 Hastings Road, Frankston, Victoria, 3199, Australia; Peninsula Health, 2 Hastings Road, Frankston, Victoria, 3199, Australia; Peninsula Health, 2 Hastings Road, Frankston, Victoria, 3199, Australia; Peninsula Health, 2 Hastings Road, Frankston, Victoria, 3199, Australia; National Centre for Healthy Ageing, Ngarnga Centre, 2 Hastings Road, Frankston, Victoria, 3199, Australia; Peninsula Clinical School, School of Translational Medicine, Monash University, 2 Hastings Road, Frankston, Victoria, 3199, Australia; Peninsula Clinical School, School of Translational Medicine, Monash University, 2 Hastings Road, Frankston, Victoria, 3199, Australia; La Trobe University, College of Science Health and Engineering, Plenty Road, Bundoora, Victoria, 3086, Australia

**Keywords:** inpatients, time and motion studies, older adults, physical activity, social participation, older people

## Abstract

**Background:**

Geriatric Evaluation and Management (GEM) services provide subacute care for older adults with complex conditions. Meaningful activities are important for preventing functional decline in hospitalised older adults; however, no studies have evaluated GEM inpatients’ participation in such activities.

**Objective:**

To determine the proportion of time GEM inpatients spend performing meaningful activities during the day and investigate whether ward environments and day of the week are associated with activity levels.

**Method:**

This observational study used behavioural mapping to audit inpatients across three GEM wards. Observations were made at 10-minute intervals over 12 consecutive hours on weekdays and 10.5 hours on weekend days. Activities were categorised as physical, cognitive or social. Logistic mixed models were used to analyse factors associated with engagement in meaningful activities.

**Results:**

In total, 60 030 minutes were observed among 70 inpatients. Overall, GEM inpatients spent 16%, 6% and 18% of observed time in physically, cognitively and socially meaningful activities, respectively. Weekend days were associated with higher odds of cognitive engagement (OR 4.79, 95% CI 1.71–13.41, *P* = .003) but lower odds of social engagement (OR 0.57, 95% CI 0.38–0.85, *P* = .006). Time spent outside patients’ rooms was positively associated with all types of meaningful activities. The odds of physically meaningful activity were not found to vary between weekends and weekdays.

**Conclusions:**

GEM inpatients demonstrate lower engagement in meaningful activities compared to rehabilitation contexts. The positive association between activity levels and time spent outside patients’ rooms emphasises the importance of accessible communal areas. Interventions to promote active participation are needed in inpatient GEM settings.

## Key Points

- This study provides the first comprehensive analysis of meaningful activity patterns among Geriatric Evaluation and Management inpatients, revealing significantly lower levels of meaningful activity compared to rehabilitation settings.
- Findings highlight the importance of communal spaces in promoting physical, cognitive and social activities.
- Given the increasing pressures on hospitals to optimise space utilisation, these results emphasise the need to balance efficient use of resources with environments that facilitate patient engagement.

## Introduction

Geriatric Evaluation and Management (GEM) provides subacute care for people with complex conditions associated with ageing [1, 2]. GEM aims to improve the functioning of a person with multidimensional needs [2] through a coordinated multidisciplinary approach [1]. In the inpatient setting, trials conducted across six countries have found that GEM reduces the risk of functional decline at discharge and reduces the risk of discharge to a residential aged care facility [1, 3]. As such, GEM services are thought to play a significant role in meeting the demand for healthcare services from an ageing population with increasing complexity of healthcare needs [4].

It is important that people receiving GEM inpatient services are engaged in meaningful activities to ensure that they do not functionally decline [5–7]. Meaningful activities include any purposeful physical movements (i.e. physical activity), verbal or nonverbal interaction with others (i.e. social activity), or engagement in nonphysical mental tasks (i.e. cognitive activity) [8–10]. These activities can be completed by inpatients as a part of the therapy they receive in GEM or outside of this therapy. Additionally, there is well-established evidence between participation in meaningful activities and the minimisation of hospital-acquired harms such as delirium, falls and pressure injuries [11]. Despite the established benefits of engaging meaningfully, participation in such activities declines with age, disability and admission to the hospital, which further highlights the importance of people receiving GEM services engaging meaningfully within an inpatient context [12, 13]. However, to date, there has been no evaluation of GEM inpatients’ participation in meaningful activities.

Research on meaningful activities has predominantly focused on inpatient rehabilitation settings. These studies have found that inpatients spend up to 27% of their time inactive [10, 14, 15], approximately half of their time performing physical activities and large variations in time spent performing cognitive (28% to 65%) and social (42%–66%) activities [8, 9, 15]. Further, prior work suggests ward environments and day of the week (i.e. weekday vs. weekend) influence inpatients’ participation in meaningful activities [9, 15, 16]. These results are not necessarily generalisable to inpatient GEM populations. GEM units differ from a general rehabilitation unit mode of service due to increased complexity of medical needs of the patients [17]; GEM units have a greater focus on geriatric medical input and longer-term care planning, compared to rehabilitation where the focus is on maximising independence for return to home [2, 18]. As such, GEM units typically have less staff than rehabilitation units, which may influence participation in meaningful activities [9].

The proposed study aims to understand the activity patterns of inpatients in three GEM wards. The findings are expected to provide a robust foundation for designing evidence-based strategies to promote active and engaged participation among older adults in GEM settings, ultimately contributing to improved health outcomes and enhanced quality of life.

The research questions were:

1. What proportion of time do GEM inpatients spend performing meaningful activities during the day?
2. Are ward environments and day of the week (i.e. weekday vs. weekend) associated with the proportion of time GEM inpatients spend performing meaningful activities?

## Method

### Design

This prospective observational study was conducted using behavioural mapping techniques [19, 20]. Data were collected between September and November 2022. Given the observational nature of the study, a waiver of patient consent was sought, and the study received institutional ethics approval from the Peninsula Health Human Research Ethics Committee prior to commencement (HREC/87339/PH-2022).

### Setting and participants

This study was conducted at a 90-bed GEM hospital. Patients are typically over the age of 65 years, admitted following an acute care admission and have a range of diagnoses including falls and related injuries, dementia and/or delirium, stroke, functional decline or infection.

The GEM hospital consists of three wards; two wards are designated as general wards, and one is a specialised ward. While the general wards share identical layouts and accessibility, the specialised ward is specifically designed to accommodate inpatients with cognitive impairments or behaviours of concern (BOCs) and has locked access points to ensure a secure environment for those at risk of wandering or absconding. Staff working on wards included the treating team (nursing, medical, allied health and healthcare assistants) and support staff (cleaners, kitchen staff, administration). Refer to Appendix 1a of the Supplementary Data section for the description of ward layout and staffing profiles.

All inpatients on the participating wards were considered for inclusion. Those being actively palliated were excluded, along with patients with planned discharge/transfer to another ward prior to data collection. A random sample of 15 inpatients was drawn using a computer random number generator on the morning of the audits, from each of the three 30-bed wards.

### Procedure

Behaviour mapping [19, 20] was used to observe patient participation in meaningful activities. This approach allows for diverse activity types to be identified and captures patients’ social interactions that are often missed using other study designs, such as accelerometry [21]. Behaviour mapping also provides important contextual information for the activities observed (e.g. location).

Meaningful activity included any purposeful physical movements (i.e. physical activity), verbal or nonverbal interaction with others (i.e. social activity) or engagement in nonphysical mental tasks (i.e. cognitive activity) [8–10]. Inactivity was, therefore, defined as the absence of any of these activities. This method of data collection required categorisation of activities as physical, social or cognitive engagement, without dual-task recording [20].

To obtain an estimate of patient activity, behaviour was audited at 10-minute intervals over 12 consecutive hours of a weekday (Monday—Friday) and 10.5 consecutive hours of a weekend day (Saturday or Sunday) between 7:00 and 17:30. Observers (i.e. data collectors) were unknown to participants, and two observers were allocated to each ward. The observer moved through each location in a pre-determined circuit, commencing a new circuit every 10 minutes. Observations on each patient were made for 15 seconds. At each observation timepoint, the category of activity (i.e. physical, cognitive, social), location, people present, clothing, footwear and equipment (i.e. aids and leisure) was recorded. To respect patient privacy, patients were not followed off-site nor intruded on if behind closed curtains or in the bathroom. Two observers were used, per ward, for all observation periods, and a third observer was used for relief periods (i.e. lunch breaks). In total, nine auditors (healthcare professionals *n* = 6; clinical researchers *n* = 3) recorded observations. Auditors received training in the auditing approach by the lead researcher to ensure accuracy. In instances where direct observations were unable to be made (e.g. while a participant was in the bathroom), activity was recorded after confirming with attending clinical staff. Where this was unable to be confirmed, time was classified as unobserved.

### Outcome measures

Time spent in activity was recorded using a purpose-designed audit form, adapted from previously published work [22–24]. The category domains can be found in Appendix 1b of the Supplementary Data section.

The audit record sheet listed options under each of the respective domains, and data were recorded as binary (yes/no). The day of audit was also recorded (i.e. weekend vs. weekday). Activity data from the recorded observations were classified as either meaningful or nonmeaningful engagement.

Participant demographics were collected to describe the cohort, including: sex, age, admission functional independence measure (FIM) score, time from admission to observation, ward (i.e. general or specialised), and reason for admission. FIM is routinely administered on the wards, and therefore, consistent with previous evaluations of GEM units was used as a measure of function and cognition [17, 25].

### Data analysis

Average time spent on tasks per day (minutes/observed time) and proportion of time spent on task categories (%) were used to describe the data. Participant characteristics were described using proportion (%) and mean (SD).

To investigate the extent to which individual factors (sex, age, presenting diagnosis, comorbidities, admission FIM score, presence of cognitive impairment, admitted ward, location, day of week and company present) were associated with engagement in meaningful activity, logistic mixed models were fit at the timepoint level to compare the odds of patients engaging (or not) in meaningful activity. Standard logistic regression modelling was inappropriate for these data due to the strong correlation across timepoints and days for individual patients [26]. Integration (estimation of log-likelihood) was achieved via mean–variance adaptive Gauss–Hermite quadrature. All analyses were completed using STATA Version 16.0 [27].

To minimise missing data regarding ‘people present’ and mitigate potential bias in subsequent analyses, informed imputations based on assumptions were made for unobserved time points. These assumptions were derived from predictable patterns of patient location and established routines in GEM settings and were determined through discussion and consensus by two experienced clinician researchers from the team. The imputations are provided in Appendix 1c of the Supplementary Data section.

## Results

In total, 71 inpatients were observed. However, one participant was excluded after data collection, as their care pathway had changed during the auditing period to active palliation. Therefore, data from a total of 70 inpatients were included in data analyses. Nineteen (27%) were observed on both a weekday and weekend day, 25 (35%) were observed on only a single weekday and 26 (37%) were observed on only a single weekend day; meaning that there were 89 days of observation (44 on a weekday, 45 on a weekend day). Participant characteristics are provided in Table 1.

**Table 1:** Participant characteristics

Total patients admitted	Overall (*n* = 70)
Sex, *n* (%)	
Male	24 (34)
Female	46 (66)
Age group, mean (SD)	81 (11)
Admission FIM score, mean (SD)	
Motor	35.9 (13.5)
Cognitive	19.3 (8.2)
Total	55.2 (17.8)
Time from admission to observation, mean (SD)	34.5 (48.2)
Ward	
General wards	45 (64)
Specialised ward	25 (36)
Day of observation, *n* (%)	
Weekday only	25 (36)
Weekend only	26 (37)
Both weekday and weekend	19 (27)
Primary reason for admission, *n* (%)	
Fall	20 (29)
Functional decline	12 (17)
Fracture	11 (16)
Stroke	5 (7)
Cognition^a^	10 (14)
Pain	4 (6)
Infection	1 (1)
Other medical conditions	7 (10)

^a^Cognition affecting ability to cope with living at home.

Observations were made for a total of 60 030 minutes. Inpatients spent 85% of their time in their own room and <1% in therapy spaces (i.e. gym, therapy room). Inpatients were alone for 66% of their time, spending 13% with the treating team and 3% with support staff. Fifty percent of their time was spent in pyjamas and 35% in day clothes. Further details on location, people present, clothing, footwear and equipment are provided in Appendix Table 1 of the Supplementary Data section.

### Meaningful activity

#### Missing data

Physical activity could not be observed/confirmed for 5190 minutes (9%); cognitive activity for 4770 minutes (8%); and social activity for 5330 minutes (9%).

#### Physical activity

Inpatients spent 16% (8780/54 840 minutes) of observed time participating in meaningful physical activity. The most-performed meaningful physical activity was self-care (4%) (Table 2). Inpatients spent <1% of observed time walking. Time spent in meaningful physical activity is detailed in Appendix Table 2 of the Supplementary Data section.

**Table 2:** Time observed for physical, cognitive and social activity

Item	Overall	Day of week		Ward	
		Weekday	Weekend	1 & 2	3
	*n* = 89	*n* = 44	*n* = 45	*n* = 59	*n* = 30
**Total observed time, min**	60 030	31 680	28 350	39 780	20 250
Activity: physical, min (%)					
IADL activity	10 (<1%)	0 (<1%)	10 (<1%)	10 (<1%)	0
Lying	18 790 (31%)	9450 (30%)	9340 (33%)	12 190 (31%)	6600 (33%)
Self-care	2220 (4%)	1500 (5%)	720 (3%)	1450 (4%)	770 (4%)
Sitting	31 670 (53%)	17 130 (54%)	14 540 (51%)	21 170 (53%)	10 500 (52%)
Standing	340 (<1%)	170 (<1%)	170 (<1%)	200 (<1%)	140 (<1%)
Therapy	470 (<1%)	440 (1%)	30 (<1%)	340 (<1%)	130 (<1%)
Transferring	330 (<1%)	210 (<1%)	120 (<1%)	240 (<1%)	90 (<1%)
Walking	560 (<1%)	300 (<1%)	260 (<1%)	210 (<1%)	350 (2%)
Wandering	450 (<1%)	340 (1%)	110 (<1%)	120 (<1%)	330 (2%)
Unobserved	5190 (9%)	2140 (7%)	3050 (11%)	3850 (10%)	1340 (7%)
Activity: cognitive, min (%)					
No cognitive activity	52 160 (87%)	28 260 (89%)	23 900 (84%)	34 310 (86%)	17 850 (88%)
Cognitive therapy	100 (<1%)	100 (<1%)	0	40 (<1%)	60 (<1%)
Computer/tablet	530 (<1%)	190 (<1%)	340 (1%)	310 (<1%)	220 (1%)
Music	1060 (2%)	410 (1%)	650 (2%)	130 (<1%)	930 (5%)
Puzzles/games	310 (<1%)	130 (<1%)	180 (<1%)	220 (<1%)	90 (<1%)
Reading	1030 (2%)	500 (2%)	530 (2%)	940 (2%)	90 (<1%)
Writing/art	70 (<1%)	40 (<1%)	30 (<1%)	70 (<1%)	0
Unobserved	4770 (8%)	2050 (6%)	2720 (10%)	3760 (9%)	1010 (5%)
Activity: social, min (%)					
Socially inactive	29 250 (49%)	15 750 (50%)	13 500 (48%)	20 210 (51%)	9040 (45%)
Being in a group	40 (<1%)	0	40 (<1%)	0	40 (<1%)
Kissing	10 (<1%)	10 (<1%)	0	0	10 (<1%)
Laughing	290 (<1%)	120 (<1%)	170 (<1%)	120 (<1%)	170 (<1%)
Passive social interaction	1910 (3%)	1430 (5%)	480 (2%)	800 (2%)	1110 (5%)
Phone call	300 (<1%)	260 (<1%)	40 (<1%)	290 (<1%)	10 (<1%)
Sleeping	15 680 (26%)	7630 (24%)	8050 (28%)	10 080 (25%)	5600 (28%)
Talking	6840 (11%)	3910 (12%)	2930 (10%)	3970 (10%)	2870 (14%)
Touch/holding hands	40 (<1%)	0	40 (<1%)	20 (<1%)	20 (<1%)
Use of phone/email	340 (<1%)	210 (<1%)	130 (<1%)	230 (<1%)	110 (<1%)
Unobserved	5330 (9%)	2360 (7%)	2970 (10%)	4060 (10%)	1270 (6%)

IADL, Instrumental Activity of Daily Living.

Regression analysis showed that neither ward nor day of the week was associated with time spent performing meaningful physical activity (Table 3). Time spent in the ward space (OR 1.48, 95% CI 1.07–2.05, *P* = .019), bathroom (OR 148.96, 95% CI 64.45–344.24, *P* < .001) or other locations (i.e. gym, offsite, outside and therapy room; OR 4.35, 95% CI 2.57–7.37, *P* < .001) had higher odds of being physically meaningful than time spent in the patient’s room. Time spent with the treating team (OR 2.67, 95% CI 2.18–3.27, *P* < .001) or with visitors (OR 1.75, 95% CI 1.21–2.53) had higher odds of being physically meaningful than time spent alone.

**Table 3:** Factors influencing time spent on physically meaningful activities (*n* = 70 cases; 5484 total time points)

Variable	Patient-related tasks 5484 total time points	
	Odds ratio (95% CI)	*P*
**Sex**		
Male	Reference group	
Female	1.05 (0.79–1.40)	.751
**Cognitive impairment**		
Absent	Reference group	
Present	0.78 (0.56–1.09)	.150
**Age** ^**a**^	1.00 (0.99–1.02)	.409
**Presenting condition**		
Fall	Reference group	
Cognitive impairment	0.91 (0.58–1.42)	.674
Functional decline	0.70 (0.45–1.09)	.114
Medical	1.01 (0.61–1.67)	.956
Fracture	1.02 (0.69–1.51)	.925
Infection	0.69 (0.30–1.61)	.392
Stroke	0.78 (0.44–1.40)	.403
Pain	0.62 (0.31–1.23)	.174
**Comorbidities** ^a^	0.92 (0.83–1.04)	.208
**Wards**		
General	Reference group	
Specialised	1.14 (0.81–1.61)	.453
**Total FIM admission score** ^a^	1.00 (0.99–1.01)	.713
**Location**		
Own room	Reference group	
Ward space	1.48 (1.07–2.05)^b^	.019
Bathroom	148. 96 (64.45–344.24)^b^	<.001
Other	4.35 (2.57–7.37)^b^	<.001
**Day of week**		
Weekday	Reference group	
Weekend	0.81 (0.63–1.05)	.111
**Company**		
Alone	Reference group	
Other patients	0.75 (0.49–1.13)	.166
Treating team/support staff	2.67 (2.18–3.27)^b^	<.001
Visitors	1.75 (1.21–2.53)^b^	.003

FIM, Functional Independent Measure. Location ‘other’ includes gym, offsite, outside and therapy room.

^a^Continuous variable (no reference group).

^b^Statistically significant.

#### Cognitive activity

Inpatients spent 6% (3100/55 260 minutes) of observed time participating in meaningful cognitive activity. The most performed meaningful cognitive activities were reading (2%) and listening to music (2%). Time spent in meaningful cognitive activity is detailed in Appendix Table 3 of the Supplementary Data section.

Regression analysis showed that time spent on weekend days had higher odds of being cognitively meaningful than time spent on weekdays (OR 4.79, 95% CI 1.71–13.41, *P* = .003) (Table 4). Time spent by patients with cognitive impairment had higher odds of being cognitively meaningful than time spent by patients with an admission diagnosis of a fall (OR 8.15, 95% CI 1.11–59.82, *P* = .039). Location (ward space: OR 2.46, 95% CI 1.48–4.10, *P* = .001) was also positively associated with time spent performing meaningful activity. Time spent with visitors had lower odds of being cognitively meaningful than time spent alone (OR 0.28, 95% CI 0.08–0.92, *P* = .037).

**Table 4:** Factors influencing time spent on cognitively meaningful activities (*n* = 70 cases; 5526 total time points)

Variable	Patient-related tasks 4780 total time points	
	Odds ratio (95% CI)	*P*
**Sex**		
Male	Reference group	
Female	2.62 (0.64–10.70)	.180
**Cognitive impairment**		
Absent	Reference group	
Present	1.10 (0.22–5.34)	.907
**Age** ^a^	0.98 (0.91–1.06)	.594
**Presenting condition**		
Fall	Reference group	
Cognitive impairment	8.15 (1.11–59.82)	.039
Functional decline	1.51 (0.24–9.63)	.664
Medical	1.36 (0.13–14.40)	.800
Fracture	0.33 (0.05–2.34)	.271
Infection	^b^	
Stroke	^b^	
Pain	1.82 (0.09–35.75)	.693
**Comorbidities** ^a^	0.89 (0.51–1.57)	.695
**Wards**		
General	Reference group	
Specialised	0.68 (0.15–3.20)	.629
**Total FIM admission score** ^a^	0.98 (0.94–1.02)	.391
**Location**		
Own room	Reference group	
Ward space	2.46 (1.48–4.10)^c^	.001
Bathroom	^b^	
Other	0.72 (0.31–1.68)	.453
**Day of week**		
Weekday	Reference group	
Weekend	4.79 (1.71–13.41)^c^	.003
**Company**		
Alone	Reference group	
Other patients	1.46 (0.87–2.45)	.148
Treating team/support staff	1.43 (0.98–2.09)	.061
Visitors	0.28 (0.08–0.92)^c^	.037

FIM, Functional Independent Measure. Location ‘other’ includes gym, offsite, outside and therapy room.

^a^Continuous variable (no reference group).

^b^No observed meaningful activity for this variable.

^c^Statistically significant.

#### Social activity

Inpatients spent 18% (9770/54 700 minutes) of observed time participating in meaningful social activity. The most performed meaningful social activities were talking (2%) and passive social interaction (3%). Inpatients spent 26% of observed time sleeping. Time spent in meaningful social activity is detailed in Appendix Table 4 of the Supplementary Data section.

Regression analysis showed that time spent on weekend days had lower odds of being socially meaningful than time spent on weekdays (OR 0.57, 95% CI 0.38–0.85, *P* = .006) (Table 5). Time spent outside of the patient’s room (ward space: OR 1.62, 95% CI 1.12–2.33, *P* = .010; other: OR 1.96, 95% CI 1.05–3.66, *P* = .036) and time spent with company (other patients: OR 21.40, 95% CI 14.76–31.05, *P* < .001; treating team/support staff: OR 24.69, 95% CI 19.27–31.63, *P* < .001; visitors: OR 270.75, 95% CI 170.66–429.55, *P* < .001) were both positively associated with meaningful social activity.

**Table 5:** Factors influencing time spent on socially meaningful activities (n = 70 cases; 5470 total time points)

Variable	Patient-related tasks 5470 total time points	
	Odds ratio (95% CI)	*P*
**Sex**		
Male	Reference group	
Female	1.63 (0.92–2.89)	.095
**Cognitive impairment**		
Absent	Reference group	
Present	0.72 (0.37–1.40)	.325
**Age** ^a^	0.99 (0.96–1.02)	.489
**Presenting condition**		
Fall	Reference group	
Cognitive impairment	1.13 (0.47–2.70)	.788
Functional decline	1.36 (0.60–3.06)	.460
Medical	0.58 (0.22–1.54)	.274
Fracture	1.02 (0.46–2.26)	.971
Infection	0.23 (0.04–1.21)	.083
Stroke	0.33 (0.10–1.10)	.072
Pain	1.48 (0.42–5.25)	.541
**Comorbidities** ^a^	0.99 (0.79–1.25)	.959
**Wards**		
General	Reference group	
Specialised	1.03 (0.53–2.00)	.935
**Total FIM admission score** ^a^	1.00 (0.99–1.02)	.476
**Location**		
Own room	Reference group	
Ward space	1.62 (1.12–2.33)^b^	.010
Bathroom	0.69 (0.40–1.19)	.183
Other	1.96 (1.05–3.66)^b^	.036
**Day of week**		
Weekday	Reference group	
Weekend	0.57 (0.38–0.85)^b^	.006
**Company**		
Alone	Reference group	
Other patients	21.40 (14.76–31.05)^b^	<.001
Treating team/support staff	24.69 (19.27–31.63)^b^	<.001
Visitors	270.75 (170.66–29.55)^b^	<.001

FIM, Functional Independent Measure. Location ‘other’ includes gym, offsite, outside and therapy room.

^a^Continuous variable (no reference group.)

^b^Statistically significant.

## Discussion

This is the first behaviour mapping study conducted in a subacute GEM setting to explore how hospitalised older adults spend their time and factors associated with participation in meaningful activity. We found that inpatients spent 16%, 6% and 18% of daytime engaged in physically, cognitively and socially meaningful activities, respectively. Results from regression analyses show that weekend day increased the odds of time spent engaged in cognitively meaningful activity; however, decreased the odds of time spent in socially meaningful activity. No differences were found between days of the week for the time spent in physically meaningful activity.

Findings from our study demonstrate a distinct pattern of GEM inpatient behaviour, with lower levels of engagement observed across physical, cognitive and social activities compared to inpatient rehabilitation contexts [8–10, 14, 15] Previous studies have indicated that inpatients of rehabilitation might spend between 28% and 65% of their time in cognitive activities and 42%–66% in social activities [8, 9, 15]. In contrast, GEM inpatients in our study engaged in cognitive and social activities well below these ranges. Similarly, while physical activities have been shown to occupy approximately half the day in inpatient rehabilitation contexts [8, 9, 15], in the GEM context, only 16% of time was spent in physical activity. Some observed discrepancies in activity levels between our study and rehabilitation contexts can be reasonably explained by differences in resourcing (e.g. staffing profiles) and complexity of care needs; however, the extent of this activity difference is concerning. The notably low engagement rates in the GEM context are of particular concern, given the well-documented risks of hospital-acquired harms that disproportionately affect hospitalised older adults [28, 29]. The findings of our study possibly highlight underutilisation of therapeutic interventions and/or strategies that facilitate engagement in meaningful activities and are known to mitigate these risks [30, 31].

Consistent with a previous evaluation in a rehabilitation context, GEM inpatients were less likely to spend time on social activities [16] during the weekend. Socialisation is dependent on the availability of staff and visitors, which is lower on weekends [16]. While it is unclear why visitors are less available on weekends, staffing is more expensive on weekends and their unavailability reduces opportunities for structured group activities that facilitate social interaction [16]. Encouragingly, increasing GEM weekend staffing levels has been shown to increase provision of therapy and participation in meaningful activity [15, 17, 25]. However, higher staffing levels have had little impact on length of hospital stay and may not be cost effective [17, 25]. Alternative solutions need to be investigated, such as incentivising visitors over the weekend and/or utilising a lower-cost healthcare assistant workforce [32].

Conversely, our results suggest that less structured time over the weekend results in greater opportunities for patients to participate in independent cognitive activities. Again, this finding is consistent with previous literature that found that inpatients of rehabilitation were more likely to spend time alone performing cognitive activities, such as reading, over the weekend [16]. This finding might also suggest that therapy on weekdays is not commonly targeted towards engaging inpatients in cognitive activities. Our results suggest that this therapy was likely targeted towards those with cognitive impairments, who were the most likely to spend their time on cognitive activities. Given the benefits of cognitive training for older adults, GEM units might consider how they can provide greater opportunities for inpatients to participate in meaningful cognitive activity [33].

The strongest predictor of engagement in any meaningful activity—whether physical, cognitive or social—was the patient being outside of their own room. This finding emphasises the critical role of communal and gym spaces in promoting patient activity. Our results align with recent research in rehabilitation settings, which demonstrated that patients spent a greater proportion of time being physically and socially active in communal spaces compared to bedrooms [8]. These findings are further supported by studies showing that communal areas can increase cognitive stimulation [34, 35]. Well-resourced communal areas for eating, socialising and completing group activities contribute to increased patient activity levels throughout the day [15, 36]. Our findings on the influence of communal spaces suggest that the application of ‘whole day rehabilitation’ principles as proposed by Kelso and colleagues [8] is a potentially relevant strategy for GEM settings. This approach could significantly enhance meaningful engagement in activities through enriched communal environments, extending the benefits observed in rehabilitation settings to subacute care for older adults. Our study thus provides some justification for maintaining and prioritising accessible, enriched communal areas, particularly in the face of pressure to repurpose such spaces for additional beds to meet the demand for healthcare services [37].

This study has limitations that must be considered. First, the research was conducted across three wards at a single healthcare organisation. GEM units are heterogeneous in their organisation and practice [3] and also likely vary in culture. This may influence the working practices of staff [38, 39] and thus opportunities for inpatients to participate in meaningful activities. Therefore, the generalisability of our results to broader populations in different healthcare settings is limited. Despite this, we have provided details about our GEM wards (staffing profiles, ward layout, patient characteristics) which allow comparisons to be made to different settings; a feature that should be included in future studies. Second, to reduce the risk of COVID-19 contamination across wards, inpatients from different wards could not co-occupy the gym. Restricted access to the gym might partly explain low physical activity levels. Thirdly, the FIM may have a ceiling effect in functional status [40] and decreased sensitivity for frail or cognitively impaired cohorts [41] so the nonsignificant association between FIM scores and participation in meaningful activity should be interpreted with caution. Last, the method of data collection required categorisation of activities as either physical, social or cognitive engagement, without dual-task recording [20]. This approach poses a limitation in recording activities that inherently involve both social and cognitive elements. For example, an interaction with a visitor, which may include therapeutic speech practice, was recorded solely as ‘social engagement’, likely explaining the very low estimated odds of time with visitors as cognitively meaningful. This methodological approach does not fully capture the cognitive demands of such interactions, potentially oversimplifying the multifaceted nature of patient activities.

## Conclusion

This study provides the first comprehensive analysis of meaningful activity patterns among GEM inpatients at a single Australian subacute hospital revealing significantly lower levels of meaningful activity compared to rehabilitation settings. Findings highlight the importance of communal spaces in promoting physical, cognitive and social activities. Staff should support patients to spend time in these shared spaces to increase opportunity for engagement in meaningful activities. These results serve as a foundation for developing evidence-based strategies to enhance meaningful activity in GEM settings. Future research should focus on implementing and evaluating interventions that optimise the use of available spaces, address weekend activity disparities and tailor activities to the unique needs of the GEM population. Our findings should also be validated in other subacute GEM settings globally, to account for variances in ward-based culture and practices.

## Supplementary Material

aa-24-2046-File002_afaf043

## Data Availability

The authors confirm that the data supporting the findings of this study are available within the article [and/or] its supplementary materials.

## References

[1] Ellis G, Gardner M, Tsiachristas A et al. Comprehensive geriatric assessment for older adults admitted to hospital. Cochrane Database Syst Rev. 2017;2017:CD006211. 10.1002/14651858.CD006211.pub3. PMC6484374 28898390

[2] Department of Health and Human Services . Geriatric Evaluation and Management [Internet]. Victoria: Department of Health and Human Services; 2022 May 17 [cited 2025 Feb 24]. Available from: http://www.health.vic.gov.au/patient-care/geriatric-evaluation-and-management.

[3] Van Craen K, Braes T, Wellens N et al. The effectiveness of inpatient geriatric evaluation and management units: a systematic review and meta-analysis. J Am Geriatr Soc. 2010;58:83–92. 10.1111/j.1532-5415.2009.02621.x. 20002509

[4] The Lancet null . Making more of multimorbidity: an emerging priority. Lancet. 2018;391:1637. 10.1016/S0140-6736(18)30941-3. 29726322

[5] Brown CJ, Friedkin RJ, Inouye SK. Prevalence and outcomes of low mobility in hospitalized older patients. J Am Geriatr Soc. 2004;52:1263–70. 10.1111/j.1532-5415.2004.52354.x. 15271112

[6] Covinsky KE, Pierluissi E, Johnston CB. Hospitalization-associated disability: “she was probably able to ambulate, but I’m not sure”. JAMA. 2011;306:1782–93. 10.1001/jama.2011.1556. 22028354

[7] Zisberg A, Shadmi E, Gur-Yaish N et al. Hospital-associated functional decline: the role of hospitalization processes beyond individual risk factors. J Am Geriatr Soc. 2015;63:55–62. 10.1111/jgs.13193. 25597557

[8] Ribbe Kelso L, Stockton K, Mahendran N et al. The influence of communal spaces on patient activity in rehabilitation: a mixed methods study. Disabil Rehabil. 2024; 46:309–21. 10.1080/09638288.2022.2160834. 36587814

[9] Rosbergen ICM, Tonello I, Clark RA et al. Does hospital design impact on patient activity levels and time spent alone? Disabil Rehabil. 2022;44:3173–80. 10.1080/09638288.2020.1861117. 33336598

[10] McKillop A, Parsons J, Slark J et al. A day in the life of older people in a rehabilitation setting: an observational study. Disabil Rehabil. 2015;37:963–70. 10.3109/09638288.2014.948968. 25113571

[11] Mudge AM, McRae P, Banks M et al. Effect of a Ward-based program on hospital-associated complications and length of stay for older inpatients: the cluster randomized CHERISH trial. JAMA Intern Med. 2022;182:274–82. 10.1001/jamainternmed.2021.7556. 35006265 PMC8749692

[12] Oh A, Gan S, Boscardin WJ et al. Engagement in meaningful activities among older adults with disability, dementia, and depression. JAMA Intern Med. 2021;181:560–2. 10.1001/jamainternmed.2020.7492. 33492334 PMC7835951

[13] Irving J, Davis S, Collier A. Aging with purpose: systematic search and review of literature pertaining to older adults and purpose. Int J Aging Hum Dev. 2017;85:403–37. 10.1177/0091415017702908. 28391702

[14] Garner J, Smith M. Activity levels of inpatients admitted to two rehabilitation units in regional hospitals: an observational study. Aust J Rural Health. 2021;29:399–407. 10.1111/ajr.12703. 34051010

[15] Scrivener K, Pocovi N, Jones T et al. Observations of activity levels in a purpose-built, inpatient. Rehabilitation Facility HERD. 2019;12:26–38. 10.1177/1937586718823519. 30727762

[16] McRae P, Bew P, Smith S et al. An observational study of physical, cognitive and social activities in rehabilitation inpatients. Australas J Ageing. 2020;39:217–24. 10.1111/ajag.12785. 32096897

[17] Farlie MK, French F, Haines TP et al. The impact of additional allied health staffing on rehabilitation outcomes at discharge from a sub-acute geriatric evaluation and management unit: a quasi-experimental, pre-post intervention study. Clin Rehabil. 2022;36:1110–9. 10.1177/02692155221095645. 35466720

[18] Department of Health and Human Services . Admitted Rehabilitation [Internet]. Victoria: Department of Health and Human Services; 2024 Oct 09 [cited 2025 Feb 24]. Available from: https://www.health.vic.gov.au/patient-care/admitted-rehabilitation.

[19] Kennedy P, Fisher K, Pearson E. Ecological evaluation of a rehabilitative environment for spinal cord injured people: behavioural mapping and feedback. Br J Clin Psychol. 1988;27:239–46. 10.1111/j.2044-8260.1988.tb00780.x. 3191303

[20] Ng CF . Behavioral Mapping and Tracking*.* In: Gifford R, ed. Research Methods for Environmental Psychology. Chichester: John Wiley & Sons, Ltd, 2016, 29–51 10.1002/9781119162124.ch3.

[21] Ariza-Vega P, Shu H, Amarasekera R et al. Older adults’ activity on a geriatric hospital unit: a behavioral mapping study. AIMS Med Sci. 2019;6:33–48. 10.3934/medsci.2019.1.33.

[22] Adey-Wakeling Z, Jolliffe L, O’Shannessy E et al. Activity, participation, and goal awareness after acquired brain injury: a prospective observational study of inpatient rehabilitation. Ann Rehabil Med. 2021;45:413–21. 10.5535/arm.21034. 35000366 PMC8743846

[23] Ekegren CL, Mather AM, Reeder S et al. Can a new ward environment and intensive allied health staffing model enhance therapeutic opportunities in trauma care? A behavioural mapping study of patients’ activities and interactions. Clin Rehabil. 2022;36:1314–23. 10.1177/02692155221107739. 35712976

[24] Kuys SS, Dolecka UE, Guard A. Activity level of hospital medical inpatients: an observational study. Arch Gerontol Geriatr. 2012;55:417–21. 10.1016/j.archger.2012.02.008. 22417401

[25] Taylor NF, Lawler K, Brusco NK et al. Saturday allied health services for geriatric evaluation and management: a controlled before-and-after trial. Australas J Ageing. 2020;39:64–72. 10.1111/ajag.12669. 31069921

[26] Diez-Roux AV . Multilevel analysis in public Health Research. Annu Rev Public Health. 2000;21:171–92. 10.1146/annurev.publhealth.21.1.171. 10884951

[27] StataCorp . *Stata Statistical Software: Release 17*. College Station, TX: StataCorp LLC, 2021.

[28] Gill TM, Allore HG, Gahbauer EA et al. Change in disability after hospitalization or restricted activity in older persons. JAMA. 2010;304:1919–28. 10.1001/jama.2010.1568. 21045098 PMC3124926

[29] Loyd C, Markland AD, Zhang Y et al. Prevalence of hospital-associated disability in older adults: a meta-analysis. J Am Med Dir Assoc. 2020;21:455–461.e5. 10.1016/j.jamda.2019.09.015. 31734122 PMC7469431

[30] Fulmer T, Mate KS, Berman A. The age-friendly health system imperative. J Am Geriatr Soc. 2018;66:22–4. 10.1111/jgs.15076. 28876455

[31] de Foubert M, Cummins H, McCullagh R et al. Systematic review of interventions targeting fundamental care to reduce hospital-associated decline in older patients. J Adv Nurs. 2021;77:4661–78. 10.1111/jan.14954. 34240755

[32] Snowdon DA, Storr B, Davis A et al. The effect of delegation of therapy to allied health assistants on patient and organisational outcomes: a systematic review and meta-analysis. BMC Health Serv Res. 2020;20:491. 10.1186/s12913-020-05312-4. 32493386 PMC7268306

[33] Butler M, McCreedy E, Nelson VA et al. Does cognitive training prevent cognitive decline? Ann Intern Med. 2018;168:63–8. 10.7326/M17-1531. 29255842

[34] Anåker A, von Koch L, Heylighen A et al. “It’s lonely”: patients’ experiences of the physical environment at a newly built stroke unit. HERD. 2019;12:141–52. 10.1177/1937586718806696. 30336696 PMC6637812

[35] Luker J, Lynch E, Bernhardsson S et al. Stroke survivors’ experiences of physical rehabilitation: a systematic review of qualitative studies. Arch Phys Med Rehabil. 2015;96: 1698–1708.e10. 10.1016/j.apmr.2015.03.017. 25847387

[36] Rosbergen IC, Grimley RS, Hayward KS et al. Embedding an enriched environment in an acute stroke unit increases activity in people with stroke: a controlled before–after pilot study. Clin Rehabil. 2017;31:1516–28. 10.1177/0269215517705181. 28459184

[37] Page B, Irving D, Amalberti R et al. Health services under pressure: a scoping review and development of a taxonomy of adaptive strategies. BMJ Qual Saf. 2023 bmjqs-2023-016686;33:738–47. 10.1136/bmjqs-2023-016686. PMC11503202 38050158

[38] Hunt J, Sanchez A, Tadd W et al. Organizational culture and performance in health care for older people: a systematic review. Rev Clin Gerontol. 2012;22:218–34. 10.1017/S0959259812000044.

[39] Jun J, Kovner CT, Dickson VV et al. Does unit culture matter? The association between unit culture and the use of evidence-based practice among hospital nurses. Appl Nurs Res. 2020;53:151251. 10.1016/j.apnr.2020.151251. 32451012

[40] Demers L, Giroux F. Validité de la Mesure de l’Indépendance Fonctionnelle (MIF) pour les personnes âgées suivies en réadapation. Can J Aging. 1997;16:626–46. 10.1017/S0714980800011004.

[41] Brosseau L, Wolfson C. The inter-rater reliability and construct validity of the functional independence measure for multiple sclerosis subjects. Clin Rehabil. 1994;8:107–15. 10.1177/026921559400800203.

