# Supplementary material for: Geriatric evaluation and management inpatients spend little time participating in physically, cognitively or socially meaningful activity: a time–motion analysis
Source: Age Ageing. 2025 Mar 4;54(3):afaf043. doi: 10.1093/ageing/afaf043 (PMC11879543; doi:10.1093/ageing/afaf043)
Supplement: aa-24-2046-File002_afaf043 [file aa-24-2046-file002_afaf043.docx]

**Title: Geriatric Evaluation and Management inpatients spend little time participating in physically, cognitively, or socially meaningful activity: A time-motion analysis**

**Supplementary Data contents list:**

Appendix 1a. GEM wards layout and staffing profile

Appendix 1b. Category domains of the audit

Appendix 1c. Imputations made to missing data of the ‘people present’ category

Appendix Table 1: Time observed for location, people present, clothing, footwear, equipment – aids, and equipment – leisure

Appendix Table 2: Time (minutes) spent participating in physically meaningful activities stratified.

Appendix Table 3. Time (minutes) spent participating in cognitive meaningful activities stratified.

Appendix Table 4. Time (minutes) spent participating in socially meaningful activities stratified.

**Appendix 1: Additional method detail**

**Appendix 1a. GEM wards layout and staffing profile**

The two general wards each contain 10 single-occupant and 10 double-occupant rooms, whereas the specialized ward has 16 single-occupant and 7 double-occupant rooms. In terms of communal spaces, the general wards have two areas separated by a hallway, while the specialized ward has four separate spaces. Outdoor access varies significantly: one of the general wards has a small garden, while the other has no outdoor space; the specialized ward has two internal courtyards as well as a rear garden. All three wards share access to two external gym spaces. The key distinctions between the specialized and general wards lie in the specialized ward’s secure design, room configuration, number of communal spaces and enhanced outdoor accessibility.

Staffing for the 90-bed GEM hospital over the audited time was 2FTE of geriatric medicine, nursing ratios of 1:5 inpatients in the morning (1:6 in the afternoon), and allied health staffing of approximately: 8.6FTE physiotherapy, 8FTE occupational therapy, 7FTE social work, 2.2FTE speech pathology, 3FTE dietetics, 4.5FTE allied health assistance. The primary difference in staffing between the general wards and the specialized ward relates to diversional therapy time, with the specialized ward allocated 14 hours of diversional therapy on a Sunday. No other allied health services are provided over a weekend.

**Appendix 1b. Category domains of the audit:**

- Physical activity (i.e., self-care, sitting, standing, lying, instrumental activities of daily living, therapy, transferring, walking, wandering)
- Cognitive activity (i.e., cognitive therapy, computer/tablet, music, no cognitive activity, puzzles/games, reading, writing/art)
- Social activity (i.e., talking, laughing, group, passive social interaction, phone call, socially inactive, sleep, touch/holding hands, kissing, use of phone/email)
- Location (i.e., bathroom, gym, outside, patient’s own room, therapy room, ward spaces),
- People present (i.e., alone, other patients, support staff, treating team, visitor, Visitor and support staff, visitor and treating team),
- Clothing (i.e., day clothes, hospital gown, naked, pajamas),
- Footwear (i.e., bare feet, shoes, socks),
- Equipment aids (i.e., bed, chair, hoist, no equipment, therapy equipment, toilet, walking aid, wheelchair)
- Equipment leisure (i.e., creative equipment, hobby materials, no equipment, radio/tablet/book/phone, television)

**Appendix 1c. Imputations made to missing data of the ‘people present’ category.**

Unobserved time in the bathroom (330mins) was assumed to be “alone”; Time in the gym (10mins) was assumed to be in the company of “treating team”; time offsite (520mins) was assumed spent with “visitors”; time outside but within the grounds of the hospital (20mins), was assumed spent with “other patients”; Unobserved time in patients’ own rooms (3,230mins) was assumed to be spent “alone”; Time in a therapy room/space (20mins) was assumed spent with “treating team”; and time ward spaces (130minutes) was assumed to be spent with “other patients”.

**Appendix Table 1**: Time observed for location, people present, clothing, footwear, equipment – aids, and equipment – leisure.

| **Item** | **Overall** | **Day of week**  **Weekday Weekend** | | **Ward 1&2 3** | |
| --- | --- | --- | --- | --- | --- |
|  | **n=89** | **n=44** | **n=45** | **n=59** | **n=30** |
| **Total Observed Time, min** | **60030** | **31680** | **28350** | **39780** | **20250** |
| Location, min (%) |  |  |  |  |  |
| Bathroom | 1740 (3%) | 1110 (4%) | 630 (2%) | 1240 (3%) | 500 (2%) |
| Gym | 400 (<1%) | 400 (1%) | 0 | 270 (<1%) | 130 (<1%) |
| Offsite | 520 (<1%) | 380 (1%) | 140 (<1%) | 380 (<1%) | 140 (<1%) |
| Outside | 460 (<1%) | 40 (<1%) | 420 (1%) | 350 (<1%) | 110 (<1%) |
| Patients own room | 50810 (85%) | 26160 (83%) | 24650 (87%) | 35590 (89%) | 15220 (75%) |
| Therapy room | 150 (<1%) | 0 | 150 (<1%) | 0 | 150 (<1%) |
| Ward spaces | 5950 (10%) | 3590 (11%) | 2360 (8%) | 1950 (5%) | 4000 (20%) |
| Unobserved | 0 | 0 | 0 | 0 | 0 |
| People Present, min (%) |  |  |  |  |  |
| Alone | 39530 (66%) | 21190 (67%) | 18340 (65%) | 27850 (70%) | 11680 (58%) |
| Other patients | 3550 (6%) | 1470 (5%) | 2080 (7%) | 540 (1%) | 3010 (15%) |
| Support staff | 1650 (3%) | 790 (2%) | 860 (3%) | 1310 (3%) | 340 (2%) |
| Treating team | 7670 (13%) | 4700 (15%) | 2970 (10%) | 4670 (12%) | 3000 (15%) |
| Visitor | 2600 (4%) | 1480 (5%) | 1120 (4%) | 1760 (4%) | 840 (4%) |
| Visitor and support staff | 70 (<1%) | 40 (<1%) | 30 (<1%) | 60 (<1%) | 10 (<1%) |
| Visitor and treating team | 700 (1%) | 520 (2%) | 180 (<1%) | 320 (<1%) | 380 (2%) |
| Unobserved |  |  |  |  |  |
| Clothing, min (%) |  |  |  |  |  |
| Day clothes | 21060 (35%) | 12770 (40%) | 8290 (29%) | 11670 (29%) | 9390 (46%) |
| Hospital gown | 4010 (7%) | 3180 (10%) | 830 (3%) | 2750 (7%) | 1260 (6%) |
| Naked | 100 (<1%) | 70 (<1%) | 30 (<1%) | 30 (<1%) | 70 (<1%) |
| Pajamas | 29830 (50%) | 13510 (43%) | 16320 (58%) | 21460 (54%) | 8370 (41%) |
| Unobserved | 5030 (8%) | 2150 (7%) | 2880 (10%) | 3870 (10%) | 1160 (6%) |
| Footwear, min (%) |  |  |  |  |  |
| Bare feet | 27110 (45%) | 13240 (42%) | 13870 (49%) | 19850 (50%) | 7260 (36%) |
| Shoes | 14500 (24%) | 8780 (28%) | 5720 (20%) | 7640 (19%) | 6860 (34%) |
| Socks (incl red grip socks) | 13020 (22%) | 7620 (24%) | 5400 (19%) | 8290 (21%) | 4730 (23%) |
| Unobserved | 5400 (9%) | 2040 (6%) | 3360 (12%) | 4000 (10%) | 1400 (7%) |
| Equipment Aids, min (%) |  |  |  |  |  |
| Bed | 33920 (57%) | 17790 (56%) | 16130 (57%) | 24450 (61%) | 9470 (47%) |
| Chair | 13290 (22%) | 7280 (23%) | 6010 (21%) | 7970 (20%) | 5320 (26%) |
| Hoist | 290 (<1%) | 220 (<1%) | 70 (<1%) | 150 (<1%) | 140 (<1%) |
| No equipment | 980 (2%) | 650 (2%) | 330 (1%) | 270 (<1%) | 710 (4%) |
| Therapy equipment | 190 (<1%) | 190 (<1%) | 0 | 140 (<1%) | 50 (<1%) |
| Toilet | 350 (<1%) | 310 (<1%) | 40 (<1%) | 160 (<1%) | 190 (<1%) |
| Walking aid | 1140 (2%) | 920 (3%) | 220 (<1%) | 730 (2%) | 410 (2%) |
| Wheelchair | 2760 (5%) | 1550 (5%) | 1210 (4%) | 1050 (3%) | 1710 (8%) |
| Unobserved | 7110 (12%) | 2770 (9%) | 4340 (15%) | 4860 (12%) | 2250 (11%) |
| Equipment Leisure, min (%) |  |  |  |  |  |
| Creative equipment | 20 (<1%) | 10 (<1%) | 10 (<1%) | 10 (<1%) | 10 (<1%) |
| Hobby materials | 240 (<1%) | 160 (<1%) | 80 (<1%) | 150 (<1%) | 90 (<1%) |
| No equipment | 35350 (59%) | 16670 (53%) | 18680 (66%) | 21430 (54%) | 13920 (69%) |
| Radio/tablet/book/phone | 730 (1%) | 520 (2%) | 210 (<1%) | 450 (1%) | 280 (1%) |
| TV on and not watching | 10200 (17%) | 6590 (21%) | 3610 (13%) | 7450 (19%) | 2750 (14%) |
| TV on and watching | 7930 (13%) | 5340 (17%) | 2590 (9%) | 5940 (15%) | 1990 (10%) |
| Unobserved | 5560 (9%) | 2390 (8%) | 3170 (11%) | 4350 (11%) | 1210 (6%) |

TV: television.

**Appendix Table 2**: Time (minutes) spent participating in physically meaningful activities stratified.

| **Variable** | **Time observed** min | **Meaningful** min (%) | **Non-meaningful**  min (%) |
| --- | --- | --- | --- |
| **Total Observed Time** | 54840* | 8780 (16%) | 46060 (84%) |
| **Sex** |  |  |  |
| Male | 18950 (35%) | 2720 (14%) | 16230 (86%) |
| Female | 35890 (65%) | 6060 (17%) | 29830 (83%) |
| **Day of week** |  |  |  |
| Weekday | 29540 (54%) | 5270 (18%) | 24270 (82%) |
| Weekend | 25300 (46%) | 3510 (14%) | 21790 (86%) |
| **Ward** |  |  |  |
| 1 &2 | 35930 (66%) | 5510 (15%) | 30420 (85%) |
| 3 | 18910 (34%) | 3270 (17%) | 15640 (83%) |
| **Cognitive Impairment** |  |  |  |
| Yes | 22010 (40%) | 3570 (16%) | 18440 (84%) |
| No | 32830 (60%) | 5210 (16%) | 27620 (84%) |
| **Diagnosis** |  |  |  |
| Cognitive | 7810 (14%) | 1380 (18%) | 6430 (82%) |
| Functional decline | 7730 (14%) | 1090 (14%) | 6640 (86%) |
| Fall | 15750 (29%) | 2710 (17%) | 13040 (83%) |
| Fracture | 10240 (19%) | 1720 (17%) | 8520 (83%) |
| Infection | 1380 (3%) | 190 (14%) | 1190 (86%) |
| Medical admission | 4710 (9%) | 810 (17%) | 3900 (83%) |
| Pain | 2550 (5%) | 320 (13%) | 2230 (87%) |
| Stroke | 4670 (9%) | 560 (12%) | 4110 (88%) |
| **Location** |  |  |  |
| Bathroom | 1460 (3%) | 1400 (96%) | 60 (4%) |
| Gym | 370 (<1%) | 350 (95%) | 20 (5%) |
| Offsite | 0 | 0 | 0 |
| Outside | 440 (<1%) | 40 (9%) | 400 (91%) |
| Patient's own room | 46660 (85%) | 5970 (13%) | 40690 (87%) |
| Therapy Room | 140 (<1%) | 0 | 140 (100%) |
| Ward spaces | 5770 (11%) | 1020 (18%) | 4750 (82%) |
| **People Present** |  |  |  |
| Alone | 38980 (71%) | 4870 (12%) | 34110 (88%) |
| Other patient(s) | 3520 (6%) | 360 (10%) | 3160 (90%) |
| Support staff | 1220 (2%) | 110 (9%) | 1110 (91%) |
| Treating team | 7470 (14%) | 2600 (35%) | 4870 (65%) |
| Unobserved | 2590 (5%) | 430 (17%) | 2160 (83%) |
| Visitor | 70 (<1%) | 20 (29%) | 50 (71%) |
| Visitor and support staff | 630 (1%) | 270 (43%) | 360 (57%) |
| Visitor and treating team | 360 (<1%) | 120 (33%) | 240 (67%) |
| **Clothing** |  |  |  |
| Day Clothes | 20580 (38%) | 3350 (16%) | 17230 (84%) |
| Hospital gown | 3700 (7%) | 700 (19%) | 3000 (81%) |
| Naked | 100 (<1%) | 90 (90%) | 10 (10%) |
| Pajamas | 29710 (54%) | 4000 (13%) | 25710 (87%) |
| Unobserved | 750 (1%) | 640 (85%) | 110 (15%) |
| **Footwear** |  |  |  |
| Bare Feet | 26450 (48%) | 3570 (13%) | 22880 (87%) |
| Shoes | 14340 (26%) | 2590 (18%) | 11750 (82%) |
| Socks | 12890 (24%) | 1990 (15%) | 10900 (85%) |
| Unobserved | 1160 (2%) | 630 (54%) | 530 (46%) |

**Appendix Table 3.** Time (minutes) spent participating in cognitive meaningful activities stratified.

| **Variable** | **Time observed** min | **Meaningful** min (%) | **Non-meaningful**  min (%) |
| --- | --- | --- | --- |
| **Total Observed Time** | 55260^*^ | 3100 (6%) | 52160 (94%) |
| **Sex** |  |  |  |
| Male | 19070 (35%) | 570 (3%) | 18500 (97%) |
| Female | 36190 (65%) | 2530 (7%) | 33660 (93%) |
| **Day of week** |  |  |  |
| Weekday | 29630 (54%) | 1370 (5%) | 28260 (95%) |
| Weekend | 25630 (46%) | 1730 (7%) | 23900 (93%) |
| **Ward** |  |  |  |
| 1&2 | 36020 (65%) | 1710 (5%) | 34310 (95%) |
| 3 | 19240 (35%) | 1390 (7%) | 17850 (93%) |
| **Cognitive Impairment** |  |  |  |
| Yes | 22090 (40%) | 1160 (5%) | 20930 (95%) |
| No | 33170 (60%) | 1940 (6%) | 31230 (94%) |
| **Diagnosis** |  |  |  |
| Cognitive | 7950 (14%) | 870 (11%) | 7080 (89%) |
| Functional decline | 7730 (14%) | 560 (7%) | 7170 (93%) |
| Fall | 15910 (29%) | 710 (4%) | 15200 (96%) |
| Fracture | 10360 (19%) | 420 (4%) | 9940 (96%) |
| Infection | 1400 (3%) | 0 | 1400 (100%) |
| Medical admission | 4710 (9%) | 420 (9%) | 4290 (91%) |
| Pain | 2540 (5%) | 120 (5%) | 2420 (95%) |
| Stroke | 4660 (8%) | 0 | 4660 (100%) |
| **Location** |  |  |  |
| Bathroom | 1450 (3%) | 0 | 1450 (100%) |
| Gym | 390 (<1%) | 0 | 390 (100%) |
| Offsite | 0 | 0 | 0 |
| Outside | 440 (<1%) | 0 | 440 (100%) |
| Patient’s own room | 46990 (85%) | 2120 (5%) | 44870 (95%) |
| Therapy Room | 150 (<1%) | 100 (67%) | 50 (33%) |
| Ward spaces | 5840 (11%) | 880 (15%) | 4960 (85%) |
| **People Present** |  |  |  |
| Alone | 39280 (71%) | 1940 (5%) | 37340 (95%) |
| Other patient(s) | 3550 (6%) | 390 (11%) | 3160 (89%) |
| Support staff | 1220 (2%) | 110 (9%) | 1110 (91%) |
| Treating team | 7520 (14%) | 570 (8%) | 6950 (92%) |
| Unobserved | 2580 (5%) | 30 (1%) | 2550 (99%) |
| Visitor | 70 (<1%) | 0 | 70 (100%) |
| Visitor and support staff | 620 (1%) | 50 (8%) | 570 (92%) |
| Visitor and treating team | 420 (<1%) | 10 (2%) | 410 (98%) |
| **Clothing** |  |  |  |
| Day Clothes | 20960 (38%) | 1780 (8%) | 19180 (92%) |
| Hospital gown | 3690 (7%) | 270 (7%) | 3420 (93%) |
| Naked | 100 (<1%) | 0 | 100 (100%) |
| Pajamas | 29820 (54%) | 1040 (3%) | 28780 (97%) |
| Unobserved | 690 (1%) | 10 (1%) | 680 (99%) |
| **Footwear** |  |  |  |
| Bare Feet | 26630 (48%) | 1000 (4%) | 25630 (96%) |
| Shoes | 14500 (26%) | 1360 (9%) | 13140 (91%) |
| Socks | 12970 (23%) | 700 (5%) | 12270 (95%) |
| Unobserved | 1160 (2%) | 40 (3%) | 1120 (97%) |

N=89 days of observation; ^*^unable to determine whether activity was cognitively meaningful for 4770 min

**Appendix Table 4.** Time (minutes) spent participating in socially meaningful activities stratified.

| **Variable** | **Time observed** (min) | **Meaningful** min (%) | **Non-meaningful**  min (%) |
| --- | --- | --- | --- |
| **Total Observed Time** | 54700* | 9770 (18%) | 44930 (82%) |
| **Sex** |  |  |  |
| Male | 19080 (3%) | 2640 (14%) | 16440 (86%) |
| Female | 35620 (7%) | 7130 (20%) | 28490 (80%) |
| **Day of week** |  |  |  |
| Weekday | 29320 (5%) | 5940 (20%) | 23380 (80%) |
| Weekend | 25380 (5%) | 3830 (15%) | 21550 (85%) |
| **Ward** |  |  |  |
| 1&2 | 35720 (7%) | 5430 (15%) | 30290 (85%) |
| 3 | 18980 (3%) | 4340 (23%) | 14640 (77%) |
| **Cognitive Impairment** |  |  |  |
| Yes | 21740 (4%) | 3860 (18%) | 17880 (82%) |
| No | 32960 (6%) | 5910 (18%) | 27050 (82%) |
| **Diagnosis** |  |  |  |
| Cognitive | 7840 (1%) | 1940 (25%) | 5900 (75%) |
| Functional decline | 7610 (1%) | 1300 (17%) | 6310 (83%) |
| Fall | 15660 (3%) | 3250 (21%) | 12410 (79%) |
| Fracture | 10250 (2%) | 1420 (14%) | 8830 (86%) |
| Infection | 1400 (<1%) | 200 (14%) | 1200 (86%) |
| Medical admission | 4690 (<1%) | 540 (12%) | 4150 (88%) |
| Pain | 2560 (<1%) | 660 (26%) | 1900 (74%) |
| Stroke | 4690 (<1%) | 460 (10%) | 4230 (90%) |
| **Location** |  |  |  |
| Bathroom | 1270 (<1%) | 280 (22%) | 990 (78%) |
| Gym | 380 (<1%) | 250 (66%) | 130 (34%) |
| Offsite | 0 | 0 | 0 |
| Outside | 440 (<1%) | 40 (9%) | 400 (91%) |
| Patient's own room | 46690 (9%) | 6820 (15%) | 39870 (85%) |
| Therapy Room | 140 (<1%) | 60 (43%) | 80 (57%) |
| Ward spaces | 5780 (1%) | 2320 (40%) | 3460 (60%) |
| **People Present** |  |  |  |
| Alone | 38920 (7%) | 1640 (4%) | 37280 (96%) |
| Other patient(s) | 3490 (<1%) | 1570 (45%) | 1920 (55%) |
| Support staff | 1220 (<1%) | 430 (35%) | 790 (65%) |
| Treating team | 7480 (1%) | 3560 (48%) | 3920 (52%) |
| Unobserved | 2580 (<1%) | 2080 (81%) | 500 (19%) |
| Visitor | 70 (<1%) | 60 (86%) | 10 (14%) |
| Visitor and support staff | 630 (<1%) | 360 (57%) | 270 (43%) |
| Visitor and treating team | 310 (<1%) | 70 (23%) | 240 (77%) |
| **Clothing** |  |  |  |
| Day Clothes | 20730 (4%) | 4370 (21%) | 16360 (79%) |
| Hospital gown | 3610 (<1%) | 1050 (29%) | 2560 (71%) |
| Naked | 70 (<1%) | 20 (29%) | 50 (71%) |
| Pajamas | 29710 (5%) | 4220 (14%) | 25490 (86%) |
| Unobserved | 580 (<1%) | 110 (19%) | 470 (81%) |
| **Footwear** |  |  |  |
| Bare Feet | 26320 (5%) | 3620 (14%) | 22700 (86%) |
| Shoes | 14360 (3%) | 3690 (26%) | 10670 (74%) |
| Socks | 12960 (2%) | 2310 (18%) | 10650 (82%) |
| Unobserved | 1060 (<1%) | 150 (14%) | 910 (86%) |

n=89 days of observation; ^*^unable to determine whether activity was socially meaningful for 5330 min.
